# A systematic review of motivational interviewing in healthcare: the potential of motivational interviewing to address the lifestyle factors relevant to multimorbidity

**DOI:** 10.15256/joc.2015.5.55

**Published:** 2015-12-28

**Authors:** Kylie J. McKenzie, David Pierce, Jane M. Gunn

**Affiliations:** ^1^Psychology Department, Ballarat Health Services and Department of General Practice, University of Melbourne, Melbourne, Victoria, Australia; ^2^Rural Health Academic Centre, University of Melbourne, Ballarat, Victoria, Australia; ^3^Department of General Practice, University of Melbourne, Melbourne, Victoria, Australia

**Keywords:** Multimorbidity, patient–provider communication, patient-centred care, motivational interviewing, systematic review

## Abstract

Internationally, health systems face an increasing demand for services from people living with multimorbidity. Multimorbidity is often associated with high levels of treatment burden. Targeting lifestyle factors that impact across multiple conditions may promote quality of life and better health outcomes for people with multimorbidity. Motivational interviewing (MI) has been studied as one approach to supporting lifestyle behaviour change. A systematic review was conducted to assess the effectiveness of MI in healthcare settings and to consider its relevance for multimorbidity. Twelve meta-analyses pertinent to multimorbidity lifestyle factors were identified. As an intervention, MI has been found to have a small-to-medium statistically significant effect across a wide variety of single diseases and for a range of behavioural outcomes. This review highlights the need for specific research into the application of MI to determine if the benefits of MI seen with single diseases are also present in the context of multimorbidity.

## Introduction

Multimorbidity is defined as the diagnosis of more than one long-term condition in one person [1]. Epidemiological research has found high prevalence rates for multimorbidity [2–4]. This is particularly true in primary care, with studies in Scotland, Australia, and the USA identifying rates of 23.2% [2], 37.1% [3], and 45.2% [5], respectively. Compared with single diseases, multimorbidity is associated with a higher demand on health services, including more primary care contacts, prescriptions, and referrals for specialized care [6–8]. Demands on patients are also higher, due to burden of illness and treatment [9]. Lack of coordination of services [9–11], inattention to patient preference [7, 11], and the prevailing single-disease focus of clinical guidelines [2, 12, 13] all add to treatment burden. Applying single-disease guidelines to multimorbidity is costly, increases polypharmacy, and involves prescription of unrealistic daily self-care activities [13, 14]. Multimorbidity is a significant health issue and effective interventions are needed [13, 15–19].

Research on multimorbidity interventions is limited [8, 19, 20]. A 2013 Cochrane Collaboration review identified only 10 randomized control trials; two of which examined specific comorbidities [19]. Expert consensus recommendations emphasize supporting behaviour change to address lifestyle factors [19, 21–23]. In Canada, Fortin and colleagues examined the association between lifestyle factors and multimorbidity in 1,196 subjects and found that smoking, a diet lacking fruit and vegetables, lack of physical activity, alcohol consumption, and excess weight, are all factors associated with an increased likelihood of multimorbidity [24]. Furthermore, the likelihood of multimorbidity increased with each additional unhealthy lifestyle factor [24]. Medication adherence may also be important, given its impact on chronic condition management [25, 26]. The World Health Organization also promotes a greater focus on patient-centred skills, highlighting communication and support for behaviour change in chronic illness [23]. With its emphasis on the individual patient and focus on health-behaviour change, Fortin and colleagues suggest that motivational interviewing (MI) may be a useful intervention for the lifestyle factors impacting on multimorbidity [24].

MI has been formally defined as “…a collaborative, goal-oriented style of communication with particular attention to the language of change. It is designed to strengthen personal motivation for and commitment to a specific goal by eliciting and exploring the person’s own reasons for change within an atmosphere of acceptance and compassion” [27]. MI is characterized by the use of communication skills, such as open questions, reflective listening to express empathy, and emphasis on patient autonomy in a clinical session [27]. First described by Miller in 1983 [28], the original application of MI was in treatment programmes for people with addictions, and subsequent studies demonstrated good clinical outcomes [29]. More recently, MI has been seen as a potentially effective intervention in physical healthcare settings [30]. This has been accompanied by an increase in the publication of primary research [31] and systematic reviews of MI [32–37]. MI has been found to have a small-to-medium effect across settings and a range of target behaviours [33, 37–40]. Lundahl and Burke [41] reviewed the findings of four meta-analyses in 2009, and found that MI was significantly more effective than no treatment, and equivalent to other treatments for a range of behaviour-change outcomes. Given the breadth of application of MI, and its patient-centred focus, further evaluation of its potential in multimorbidity care is warranted.

This systematic review identifies research papers of MI in healthcare where authors have used systematic review methodology to identify primary intervention trials and have also conducted a meta-analysis. This is the first systematic review of the literature to specifically examine meta-analyses. This systematic review has three objectives. Firstly, we will examine the evidence for MI in healthcare and specifically for multimorbidity, including the effectiveness of MI for addressing the lifestyle factors relevant to multimorbidity. Secondly, given the widespread impact of multimorbidity on the healthcare system and the recommendation to integrate multimorbidity intervention into existing healthcare [19], we will examine whether MI can be delivered effectively by a range of healthcare providers. Finally, based on this analysis of the reviews, we will consider and discuss the potential of MI in clinical work of patients with multimorbidity.

## Methods

Our systematic review was guided by the Preferred Reporting Items for Systematic Reviews and Meta-Analyses (PRISMA) Statement [42, 43]. Review criteria were outlined *a priori*. 

### Inclusion criteria

Articles were included if the authors used systematic review methodology to identify relevant primary interventions, and also conducted a meta-analysis of the data from the identified primary interventions. Reviews were only included if participants were recruited from healthcare services, not criminal justice, education, or other sectors. We included reviews that identified studies of MI intervention, where authors of the reviews defined MI according to the general principles outlined by Miller and Rollnick [27], and used these principles in selecting the primary intervention papers. Included reviews were those that compared MI intervention with control, treatment as usual, or other intervention with behaviour change or standardized outcome measures. 

### Search strategy and article selection

The search included articles published up to and including January 2014. Due to practical constraints, selection was limited to English-language articles that were peer reviewed and published in full. The following electronic databases were searched: PsycInfo, Medline, CINAHL, EMBASE, and Cochrane library. In addition, we searched the online bibliography accompanying the 2013 Miller and Rollnick textbook [44]. Search terms were ‘motivational interview*’ AND [‘systematic review’ OR ‘meta-analysis’]. Terms included both subject index terms and free text. Duplicate articles were removed using the duplicate identification function in EndnoteX5 (Thomson Reuters, New York, Version X5 for Macintosh and Windows, 2011).

The search strategy and initial screening of article titles was performed by K.J.M.; articles clearly not meeting eligibility criteria were excluded. Abstracts were reviewed to determine whether a publication met the criteria for a systematic review or meta-analysis and if MI was an intervention included in the analysis. Full-text articles were reviewed to confirm eligibility. Uncertainty about inclusion of articles was resolved through discussion with the review team at regular meetings.

### Assessment of quality of systematic reviews

All included articles were reviewed by K.J.M. using AMSTAR (A MeaSurement Tool to Assess systematic Reviews [45]). AMSTAR is a reliable 11-item tool for assessing the methodological quality of systematic reviews [46]. AMSTAR items include the design and conduct of the systematic review, the presentation of review data, the scientific quality of the methods for formulating conclusions, publication bias, and conflict of interest. A score of 1 is allocated to each item that fully meets the specified criteria for each of the 11 AMSTAR items. A score of 0 is allocated if the item is not met or if there is insufficient information presented in the review article to meet the criterion. The highest score possible using AMSTAR is 11, with the high scores being indicative of better methodology [46].

### Data extraction

Effect size data were extracted for the overall efficacy of MI, as well as for alcohol, smoking cessation, diet and exercise, medication adherence, and weight interventions. Interpretation of effect sizes was guided by the benchmarks suggested by Cohen [47]. Information about the health conditions included in each review was extracted from summary tables and a review of the titles of trials included in each meta-analysis. Information about clinician type, MI training, and treatment integrity was also extracted. Data extraction was undertaken by K.J.M. with uncertainty resolved through discussion with the review team. Accuracy of data extraction was checked independently in regular formal meetings between K.J.M. and D.P., and K.J.M. and J.M.G.

## Results

### Selection of systematic reviews

**Figure 1:**
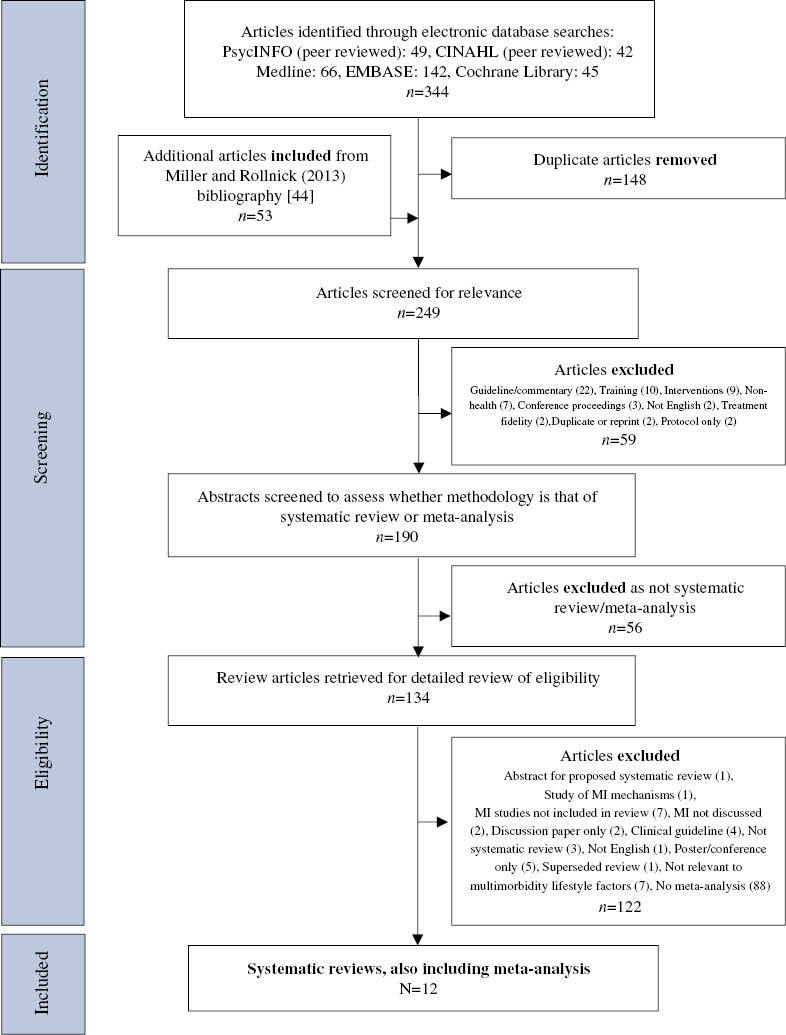
Flowchart of selection process for included articles using the following electronic databases: PsycINFO (database of abstracts produced by the American Psychological Association), CINAHL (Cumulative Index of Nursing and Allied Health Literature), Medline (Medical Literature Analysis and Retrieval System Online), EMBASE (Excerpta Medica dataBASE), Cochrane Library, and the bibliography by Miller and Rollnick [44].

The selection process is summarized in Figure 1. Twelve articles met the inclusion criteria.

### Assessment of quality of systematic reviews

**Figure 2:**
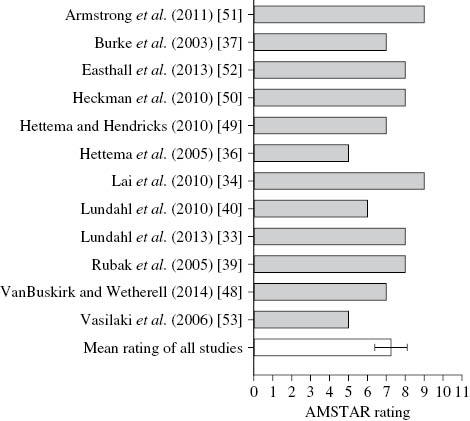
Rating for each of the identified systematic reviews that also included meta-analysis, using AMSTAR (A MeaSurement Tool to Assess systematic Reviews) [45].

The included articles were assessed using AMSTAR (see Figure 2) [33, 34, 36, 37, 39, 40, 48–53]. The mean AMSTAR rating was 7.25 (SD=1.36). None of the 12 articles met the criteria for items 5 [*Was a list of studies* *(included and excluded) provided?*] and 11 (*Was the conflict of interest stated?*). In addition, the criteria were only met for 5 of 12 studies for item 2 (*Was there duplicate study selection and data extraction?*) and 6 of 12 studies for item 4 [*Was the status of the publication (i.e. grey literature) used as an inclusion criterion?*].

### Systematic review characteristics and effect sizes

The characteristics of the included systematic reviews are summarized in Table 1 [33, 34, 36, 37, 39, 40, 48–53].

Table 2 summarizes the chronic conditions specified for participant groups in included systematic reviews [33, 34, 36, 37, 39, 40, 48–53]. Participant groups included people living with a range of conditions; however, no systematic review specifically examined multimorbidity. 

Table 3 summarizes the effect sizes, limitations, and conclusions for each systematic review [33, 34, 36, 37, 39, 40, 48–53]. Small-to-medium statistically significant effect sizes were reported for the overall effect of MI intervention across a range of health behaviours relevant to multimorbidity. Overall effect sizes ranged from *d*=0.18 [95% confidence interval (CI) 0.01, 0.37] [48] to *d*=0.77 (95% CI 0.35, 1.19) [36].

### Clinicians delivering MI interventions

Summary information about clinicians delivering MI interventions for each of the 12 meta-analyses is presented in Table 4 [33, 34, 36, 37, 39, 40, 48–53]. 

### Treatment fidelity and MI training

Table 5 presents a summary of the minimal information available for MI training and treatment fidelity [33, 34, 36, 37, 39, 40, 48–53]. 

## Discussion

### Summary of main findings

We identified 12 systematic reviews that also included meta-analysis for MI in healthcare. We did not identify a study specifically examining MI as an intervention for multimorbidity. It appears that MI is as effective as other treatments for each of the lifestyle factors relevant to multimorbidity, and that it can be delivered by a range of healthcare providers. The extent to which these findings apply to the setting of multimorbidity has yet to be determined. 

### Strengths and limitations

Overall, the included systematic reviews were of a good quality. Similar to other studies [55, 56], some items on the AMSTAR tool were not met by any publication. Items requiring more extensive statements may be affected by publication parameters. This review has synthesized significant amounts of information, and the quality of the reviews supports the conclusions drawn.

This review is limited by the fact that included reviews evaluate the effectiveness of MI for single diseases. We have examined the evidence for the lifestyle factors relevant to multimorbidity, in the absence of specific multimorbidity studies. We are therefore inferring from the available evidence about the potential of MI for multimorbidity; in particular, its potential to address lifestyle factors impacting on the health of patients with multimorbidity. 

Additionally, a potential limitation of this review is that selection was limited to English-language publications. In this case, publication bias may be ameliorated by statistical assessment of publication bias in 75% of the included systematic reviews, and searching of grey literature in 50%. There was also a lack of information about cost-effectiveness. The systematic review by Lai and colleagues identified two trials that reported information about cost, but the information was insufficient to draw any conclusions [34]. While some authors of the included systematic reviews suggested that MI may be more cost-effective than other interventions as it is a briefer intervention [40, 50, 53], the need for specific cost–effectiveness analyses was identified as an important consideration in future research [37, 40, 53]. 

### Relating the findings to the existing literature

The lack of evidence for the application of MI to multimorbidity intervention is not an unexpected finding. The Cochrane Review undertaken by Smith and colleagues [19] only identified 10 randomized controlled trials of intervention for multimorbidity and none of these included MI. Despite the lack of intervention trials, expert consensus recommendations identify patient-centred care and communication skills, promoting healthy behaviours, and integrating intervention into routine healthcare as core elements for multimorbidity intervention [4, 12, 19, 23, 24]. Indeed, some of the authors of the included reviews propose implementing MI as an intervention in routine healthcare [33, 36, 39, 40, 48, 52] and for the multiple behaviour-change challenges inherent in primary care practice [48]. 

### Implications for research and clinical practice

MI is a well-articulated and learnable skill [57–59] and appears to be a useful intervention for a range of health-behaviour-change targets, such as diet and exercise, weight management, smoking cessation, medication adherence, and alcohol consumption. All of these behaviours are relevant to people living with multimorbidity.

Further research may benefit from a greater focus on clinician proficiency, and a greater emphasis on the effectiveness of MI when delivered by a range of clinicians. Future research also needs to include treatment fidelity measures [37] to ensure the intervention being studied is indeed MI. In addition, it may also be helpful to use treatment fidelity measures with treatment as usual or comparison conditions to evaluate the degree to which MI can be differentiated from baseline communication styles in routine healthcare delivery [60].

## Conclusion

Multimorbidity presents significant challenges to the people who are living with multiple conditions and healthcare professionals alike. MI appears to be a helpful approach to healthcare across a range of single diseases, and for health-behaviour change. Based on the existing recommendations for multimorbidity interventions and the findings of this review, it appears that research that directly examines the application of MI for working with people with multimorbidity is warranted. 

## Figures and Tables

**Table 1:** Summary of characteristics of the included systematic reviews.

Reference	Date	No. of publications identified in systematic review	No. of trials included in analyses	Range of years of included studies	Median year of publication of included studies	Author/s country of origin	Total no. participants (included in analyses)	Target behaviour/outcome
Armstrong *et al*. [51]	2011	11	12	1995–2009	2007	Canada	2,938	Weight loss
Burke *et al*. [37]	2003	30	30	1988–2001	1998	USA	6,385	Multiple behaviours^a^
Easthall *et al*. [52]	2013	26	26	1990–2012	2005	UK	5,216	Medication adherence
Heckman *et al*. [50]	2010	31	31	1998–2009	2005	USA	9,485	Smoking cessation
Hettema and Hendricks [49]	2010	31	31	1998–2009	2005	USA	8,165	Smoking cessation
Hettema *et al*. [36]	2005	72	72	1992–2004	2000	USA	14,267	Multiple behaviours^b^
Lai *et al*. [34]	2010	14	14	1997–2008	2005	Hong Kong, UK, China	10,538	Smoking cessation
Lundahl *et al*. [40]	2010	119	132	1989–2007	2004	USA	17,173	Multiple behaviours^c^
Lundahl *et al*. [33]	2013	48	51	1997–2011	2007	USA/UK	9,618	Multiple behaviours^d^
Rubak *et al*. [39]	2005	72	19	1988–2002	1998	Denmark	Not stated	Multiple behaviours^e^
VanBuskirk and Wetherell [48]	2014	12	12	2001–2011	2008	USA	3,326	Multiple behaviours^f^
Vasilaki *et al*. [53]	2006	15	9: c/f No Tx 9: c/f Other Tx	1988–2003	1999	UK	2,767	Alcohol reduction

c/f, compared with; Tx, treatment.

^a^Alcohol (15), diet and exercise (4), drug use (5), HIV-risk (2), eating disorder (1), smoking (2), treatment adherence (1).

^b^Alcohol (29), blood pressure (1), diet (2), drug use (14), eating disorder (1), HIV-risk (5), gambling (1), medication adherence (1), public health intervention (1), smoking (6), treatment adherence (4), weight (1), not specified (1).

^c^Alcohol (3), blood pressure (2), drug use (1), health promotion (3), smoking (2), not specified (1).

^d^Alcohol (39), breastfeeding (1), dental health (1), diabetes management (1), diet (2), drug use (23), HIV-risk (4), gambling (2), health promotion (7), medication adherence (2), physical activity (3), public health intervention (1), smoking (17), treatment adherence (10), weight (1) not specified (3).

^e^Alcohol (6), breastfeeding (1), dental health (2), diabetes management (4), diet (3), diet and exercise (1), drug use (3), Functional independence (2), eating disorder (1), HIV-risk (2), injury prevention (2), medication adherence (3), physical activity (1), quality of life (2), safe sex (1), self-management (3), smoking (8), treatment adherence (1), weight (2).

^f^Alcohol (3), diet and exercise (1), drug use (2), medication adherence (1), smoking (2), smoking, diet, and exercise (1), treatment adherence (1), weight (1).

**Table 2:** Chronic conditions specified for participant groups in included motivational interviewing (MI) trials, determined by summary information and title search.

Reference	Date	No. of MI publications included in meta-analysis	Asthma	Cancer	Cardiac condition	COPD	Diabetes	Epilepsy	GI	HIV	Hyperlipidaemia	Hypertension	MS	Osteoporosis	Pain	Psychiatric illness	Stroke	Not specified
Armstrong *et al*. [51]	2011	11					3				1	1						6
Burke *et al*. [37]	2003	30					1				1	1				3		24
Easthall *et al*. [52]	2013	11					1			6		2	1	1				0
Heckman *et al*. [50]	2010	31 (30 listed)		1			1			1		1				2		24
Hettema and Hendricks [49]	2010	31		1		1	2			1		1				2		23
Hettema *et al*. [36]	2005	72 (68 listed)					1			5	1	1				10		50
Lai *et al*. [34]	2010	14		1	1		1											11
Lundahl *et al*. [40]	2010	119 (118 listed)	1		1		2		1	2						10	1	100
Lundahl *et al*. [33]	2013	48		1	2		4	1		4	1				2	2	2	29
Rubak *et al*. [39]	2005	19					4				2	1						12
VanBuskirk and Wetherell [48]	2014	12					1					2						9
Vasilaki *et al*. [53]	2006	15																15
No. of unique references identified for each chronic condition type			1	3	3	1	12	1	1	15	2	4	1	1	2	16	2	

COPD, chronic obstructive pulmonary disease; GI, gastrointestinal; HIV, human immunodeficiency virus; MS, multiple sclerosis.

**Table 3:** Summary of effect sizes, limitations, and conclusions for included meta-analyses.

Reference	Overall	Alcohol	Smoking	Diet and exercise	Medication adherence	Weight	Limitation	Conclusion
Armstrong *et al*. (2011) [51]	–	–	–	–	–	*d*=−0.51 [−1.04, 0.01] (*k*=12, NT:9, AC:2); *N*=1,448; 11 references^a^; 8 unique^b^	Low statistical power. Publication bias likely	MI offers a useful adjunct intervention to current interventions.
Burke *et al*. (2003) [37]	–	*d*=0.25* [0.13, 0.37] (*k*=12, NT: 12); *N*=1,142; 9 references^a^; 0 unique^b^	*d*=0.11 [−0.05, 0.27] (*k*=2, NT:2); *N*=574; 2 references^a^; 0 unique^b^	*d*=0.53* [0.32, 0.74] (*k*=4, NT: 4); *N*=366; 4 references^a^; 1 unique^b^	–	–	Search limited to PsycINFO and trainer network	Adaptations of MI as effective as other active treatments, in a shorter time frame
Easthall *et al*. (2013) [52]	–	–	–	–	*g*=0.14 [−0.067, 0.341] (*k*=11, SC:10, MC: 1); *N*=3,739; 11 references^a^; 10 unique^b^	–	Effect sizes corrected for publication bias	MI efficacious but not superior to other medication adherence interventions
Heckman *et al*. (2010) [50]	–	–	OR=1.45* [1.14, 1.83] (*k*=31, VC); *N*=9,485; 3 references^a^; 10 unique^b^	–	–	–	Slight publication bias/fidelity not well assessed/mainly US studies	MI efficacious for smoking cessation in adolescents and adults, but not for perinatal women
Hettema and Hendricks (2010) [49]	–	–	*d*=0.12 ^NP S^ [−0.05, 0.28] (*k*=16, VC); N=6,437; *d*=0.17* ^NP L^ [0.01, 0.32] (*k*=21, VC); *N*=8,210; 23 references^a^; 8unique^b^	–	–	–	Few studies in this meta-analysis examine MI on its own, fidelity poorly reported in included studies	MI has some efficacy for smoking cessation
Hettema *et al*. (2005) [36]	*d*=0.77* [0.35, 1.19] (*k*=72, VC); *N*=14,267; 68 listed references^a^; 33 unique^b^	*d*=0.26* [0.18, 0.33] (*k*=31, VC); *N*=6,180; 31 references^a^; 18 unique^b^	*d*=0.14* [0.09, 0.20] (*k*=6, VC); *N*=2,203; 6 references^a^; 0 unique^b^	*d*=0.78* [0.41, 1.16] (*k*=4, VC); *N*=851; 4 references^a^; 1 unique^b^	*d*=0.72* [0.56, 0.89] (*k*=5, VC); *N*=374; 5 references^a^; 5 unique^b^	-	Search limited to PsycINFO and trainer network. Publication bias not assessed	MI appears a useful stand-alone intervention, with additive potential to other interventions
Lai *et al*. (2010) [34]	–	–	RR=1.27* [1.14, 1.42] (*k*=14, BA or SC); *N*=10,538; 14 references^a^; 6 unique^b^	–	–	–	Trial quality, fidelity and reporting bias may impact	MI appears moderately successful for smoking cessation
Lundahl *et al*. (2010) [40]	*d*=0.22* [0.17, 0.27] (*k*=132, AC: 1, SC: 81, WL: 35, IO: 10)^c^; *N*=17,173; 119 references^a^; 72 unique^b^	–	–	–	–	–	Publication bias not assessed	MI has application across a range of health outcomes
Lundahl *et al*. (2013) [33]	OR=1.55* [1.4, 1.71] (*k*=51, WL: 7, IO: 16, SC: 28); *N*=9,618; 48 references^a^; 28 unique^b^	–	–	–	–	–	Limited information about fidelity, difficult to ascertain comparison conditions	MI efficacious in medical settings for some target behaviours
Rubak et al (2005)^d^ [39]	–	14.64* units alcohol/week [13.73, 15.55] (*k*=7, TAG: 7); 72.92 mg%* [46.80, 99.04] (*k*=6, TAG: 6); 8 references^a^; 1 unique^b^	1.32 cigs/day [−0.25, 2.88] (*k*=3, TAG:3); 3 references^a^; 0 unique^b^	–	–	0.72 BMI or kg/m^2*^ [0.33,1.11] (*k*=6, TAG: 6); 6 references^a^; 5 unique^b^	Data not reported as effect sizes, so comparison is more difficult. Small number of studies for smoking	MI outperforms traditional advice giving
VanBuskirk and Wetherell (2014) [48]	*d*=0.18* [0.01, 0.37] (*k*=16, VC); *N*=3,326; 12 references^a^; 7 unique^b^	*d*=0.22 [−0.21, 0.65] (*k*=6, includes smoking, alcohol and other drugs, VC); *N*=1,504; 4 references^a^; 4 unique^b^	–	Physical activity only, *d*=0.07 [−0.08, 0.21] (*k*=3, SC: 2, SC+I: 1); *N*=764; 3 references^a^; 3 unique^b^	*d*=0.19* [0.01, 0.37] (*k*=2, SC: 2), *N*=794; 2 references^a^; 1 unique^b^	*d*=0.47 [−0.04, 0.99] (*k*=2, SC: 1, SC+I: 1); *N*=475; 2 references^a^; 2unique^b^	Subgroup meta-analyses lacked power due to small sample sizes. Data for smoking, alcohol and other drugs combined	Support for the application of MI in primary care settings for range of behaviours
Vasilaki *et al*. (2006) [53]	–	*d*=0.18* [0.07, 0.29] (*k*=9, NT: 9); *d*=0.43* (*k*=9, AT: 9) [0.17, 0.70]; *N*=2,767; 15 references^a^; 4 unique^b^	–	–	–	–	Fixed-effects model used; however, significant heterogeneity	Brief MI effective for reducing excessive drinking
Combined total no. of references cited^e^	191	40	46	8	17	16		

[…]: 95% confidence intervals; –, no effect size provided; AC, attention control; AT, active treatment; BA, brief advice; BMI, body mass index; cigs, cigarettes; *d*, Cohen’s *d*; *g*, Hedges’ *g*; I, information; IO, information only; *k*, no. of included trials; L, longterm follow-up;. MC, minimal contact; NP, non-pregnant sample; NT, no treatment; OR, odds ratio; RR, risk ratio; S, short-term follow-up; SC, standard care; TAG, traditional advice giving; VC, various comparison conditions; WL, waiting list.

*Statistically significant.

^a^Number of references included in this meta-analysis.

^b^Number of references included in this meta-analysis and not in the other listed meta-analyses.

^c^Data provided for 127 trials only.

^d^Sample size data not provided.

^e^Total number of references cited across meta-analyses, including those that were not unique to any one review.

**Table 4:** Summary of available data about clinician type and effect of clinician type in each systematic review.

Reference	No. of studies in analysis	Clinicians (*n*)	Effect of clinician type
Armstrong *et al*. (2011) [51]	11	Nurse (2), psychologists (2), dietician (1), dietician/physical activity specialist (1), psychology students (2), counsellor (1), health promotion counsellors (1), exercise scientists (1)	Not reported
Burke *et al*. (2003) [37]	30	Not reported	Not reported
Easthall *et al*. (2013) [52]	26	Specialist (2), researcher ( 3), routine HCP (4), nurse (1), health educator (1)	No effect of clinician type (across MI and other behaviour-change techniques)
	11 MI studies		
Heckman *et al*. (2010) [50]	31	36% counsellors/therapists, 18% staff/interventionists, 12% nurses/midwives, 9% mixed, 6% psychologists, 6% physicians, 6% health educators and 6% trainees	No effect of clinician type
Hettema and Hendricks (2010) [49]	31	Mental health and medical providers	Not reported
Hettema *et al*. (2005) [36]	72	Paraprofessionals or students (8), Master’s level counsellors (6), psychologists (6), nurses (3), physicians (2), dieticians (1), and varying levels of professionals (22)	Not reported
Lai *et al*. (2010) [34]	14	Primary care physicians (2), hospital physicians (2), nurses (4), counsellors (8), psychologists (1)	Effective when delivered by primary care physicians and by counsellors
Lundahl *et al*. (2010) [40]	119	Mental health (Bachelors): (8), mental health (Masters/PhD) (12), nurse (5), student (6)	No effect of clinician type
Lundahl *et al*. (2013) [33]	48	Dietician (3), physician (2), mental health providers (13), mixed (9), nurse (6)	All provider types produced positive outcomes with statistically significant effects for mixed team and mental health providers
Rubak *et al*. (2005) [39]	72	Psychologist (42), doctor (23), HCP (including nurse, midwife, dietician) (11)	Effect obtained by 83% of physician studies, 80% of studies with psychologists and 46% of studies with other HCPs 46%
Vasilaki *et al*. (2006) [53]	15	PhD student (3), student (6), clinician (4), nurse (1), staff (1)	Not reported
VanBuskirk and Wetherell (2014) [48]	12	Physicians or nurse practitioners (3), Master’s level therapist (1), health educator/counsellor/research assistant (8)	Higher qualifications associated with significantly better outcomes for substance use, and overall

HCP, healthcare providers; MI, motivational interviewing.

**Table 5:** Summary of motivational interviewing training and treatment fidelity measures in each systematic review.

Reference	No. of studies in analysis	MI training	Studies providing MI training information (%)	Treatment fidelity	Studies providing treatment fidelity information (%)
Armstrong *et al*. (2011) [51]	11	Not reported	n/a	7 reported a measure of fidelity	64
Burke *et al*. (2003) [37]	30	Authors note most included trials did not sufficiently describe training.	n/a	Not well described	n/a
Easthall *et al*. (2013) [52]	26	Not reported	n/a	Not reported	n/a
Heckman *et al*. (2010) [50]	31	11/31 studies. Mean 52 hours (SD 72)	36	17 reported a measure of fidelity	55
Hettema and Hendricks (2010) [49]	31	16/23 studies mentioned MI training; 7 studies reported training hours. Mean 28.14 hours (SD 25.89); range: 2–75 hours	70	11 reported post-training supervision/support5 reported competency assessment 3 reported some form of monitoring	61
Hettema *et al*. (2005) [36]	72	13/72 studies. Mean 9.92 hours (SD 7.35)	18	Not reported	n/a
Lai *et al*. (2010) [34]	14	11/14 studies; 2–12 hours workshop training	79	3 reported audio recording; 4 reported supervision; 1 reported booster training; 1 reported support meeting; 1 reported use of MISC	71
Lundahl *et al*. (2010) [40]	119	Not reported	n/a	43 reported no assessment; 22 reported qualitative assessment; 17 reported standardized assessment	33
Lundahl *et al*. (2013) [33]	48	24/48 studies. Mean 18 hours (range 4–40)	50	8 reported a measure of fidelity	17
Rubak *et al*. (2005) [39]	19	Not reported	n/a	Not reported	n/a
VanBuskirk and Wetherell (2014) [48]	12	5/12 studies. 8 hours to 4 weeks training	42	6 reported supervision	50
Vasilaki *et al*. (2006) [53]	15	Not reported	n/a	4 reported a measure of fidelity	27

MISC, motivational interviewing skills code; a coding system for motivational interviewing, see Moyers *et al*. [54].

n/a, not available.
